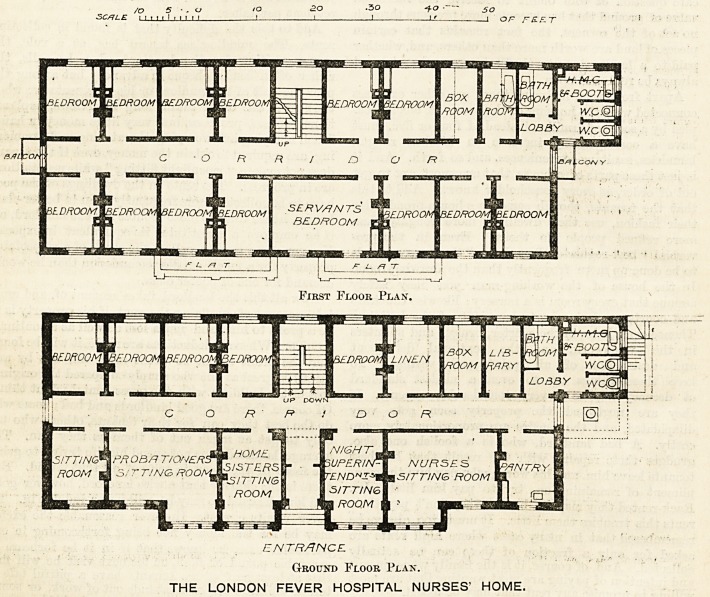# Hospital Construction

**Published:** 1899-05-06

**Authors:** 


### The Institutional Workshop.

### HOSPITAL CONSTRUCTION.

### THE LONDON FEVER HOSPITAL NURSES' HOME.

This nurses' home is arranged on a very simple plan; a straight corridor running from end to end of the building with rooms on each side, the several floors being connected by a staircase abutting on the centre of the corridor. On the ground floor are sitting-rooms for the home sister, the night superintendent, the nurses,

First Floor Plan.

Ground Floor Plan.

THE LONDON FEVER HOSPITAL NURSES' HOME.

and the probationers, together with library, box-room, linen-room, pantry, bath-room, lavatory, &c., and several bedrooms. Upstairs are bedrooms. The closets are placed in a projecting block at one end of the building, from which they are properly cut off. By the introduction of a box-room between the bath-room and the next bedroom the latter is saved the noise and discomfort which would otherwise have been entailed upon its occupant. Each end of the corridor of the upper floor opens on a small balcony, which might easily have been arranged to provide an emergency staircase.

## Figures and Tables

**Figure f1:**